# Penile Squamous Cell Carcinoma in a 95-Year-Old Patient: Case Report and Literature Review

**DOI:** 10.7759/cureus.32660

**Published:** 2022-12-18

**Authors:** Wassim Alaoui Mhammedi, Anouar El Moudane, Mohamed Irzi, Mohamed Mokhtari, Ali Barki

**Affiliations:** 1 Urology, Mohammed VI University Hospital, Oujda, MAR

**Keywords:** penile neoplasms, carcinoma, squamous cell carcinoma, penis, penile cancer

## Abstract

Penile cancer is a rare disease. Delay in diagnosis and treatment frequently occurs and high morbidity can be observed in affected patients. The management is based on surgical resection. The inguinal node staging depends on the disease-related risk. We herein report a 95-year-old male patient, with a history of a right inguinal hernia, surgically treated. He presented with a swollen painful glans penis. The glans biopsy identified a moderately differentiated invasive squamous cell carcinoma. We have performed a complete penectomy with a bilateral inguinal lymph node dissection. The patient is currently receiving adjuvant chemotherapy based on cisplatin

## Introduction

Penile cancer is a rare disease with an incidence of one affected man in 100,000 worldwide every year [1]. Histologically, squamous cell carcinoma (SCC) is the most reported cancer type in the penis [1]. In this article, we report a 95-year-old male patient, with a history of a cured right inguinal hernia. He presented with a swollen painful glans. Biological investigation revealed no abnormalities. The glans biopsy identified a moderately differentiated invasive squamous cell carcinoma. We have performed a complete penectomy with bilateral inguinal lymph node dissection. The patient is currently receiving adjuvant chemotherapy based on cisplatin.

## Case presentation

**Figure 1:**
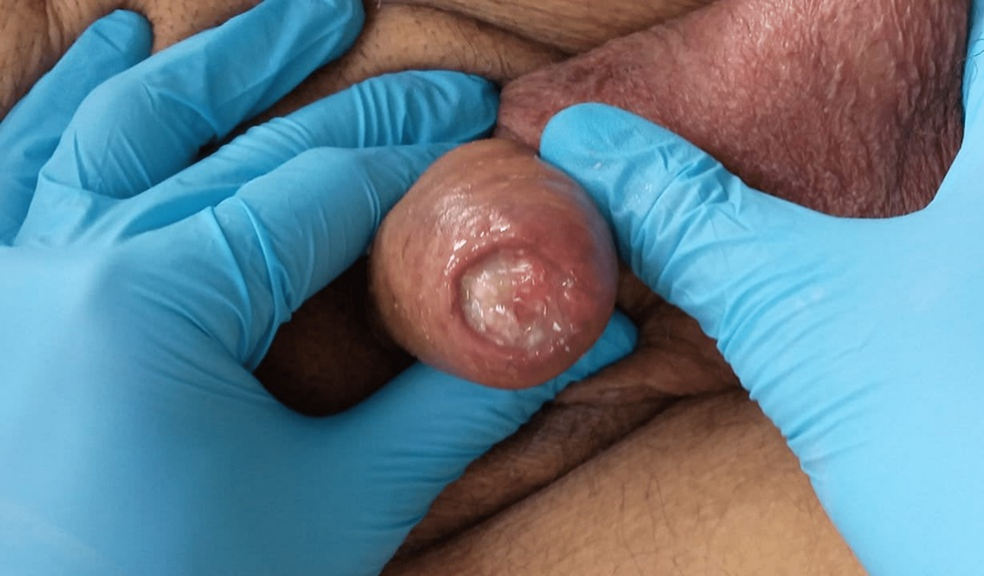
Examination of the penis revealing a 1.3 x 1 cm ulcerated lesion of the gland The surrounding glans’ skin was erythematous with no extension to the foreskin.

We report a 95-year-old male patient, with a history of a right inguinal hernia that was treated surgically. No cigarette smoking and no multiple sexual partners are reported. The patient was not circumcised. He presented with a history of 2 months of glans swelling and pain. Clinical evaluation revealed a stable patient with a mildly high arterial blood pressure at 145/93 mmHg, a normal respiratory rate at 16 breaths per minute, and a normal heart rate of 98 beats per minute. Examination of the penis revealed a 1.3 x 1 cm ulcerated lesion of the glans. The surrounding skin was erythematous with no extension to the foreskin. No phimosis was observed. Palpation identified a firm base of the visible lesion. No lymphadenopathies were identified clinically (Figure 1).

**Figure 2:**
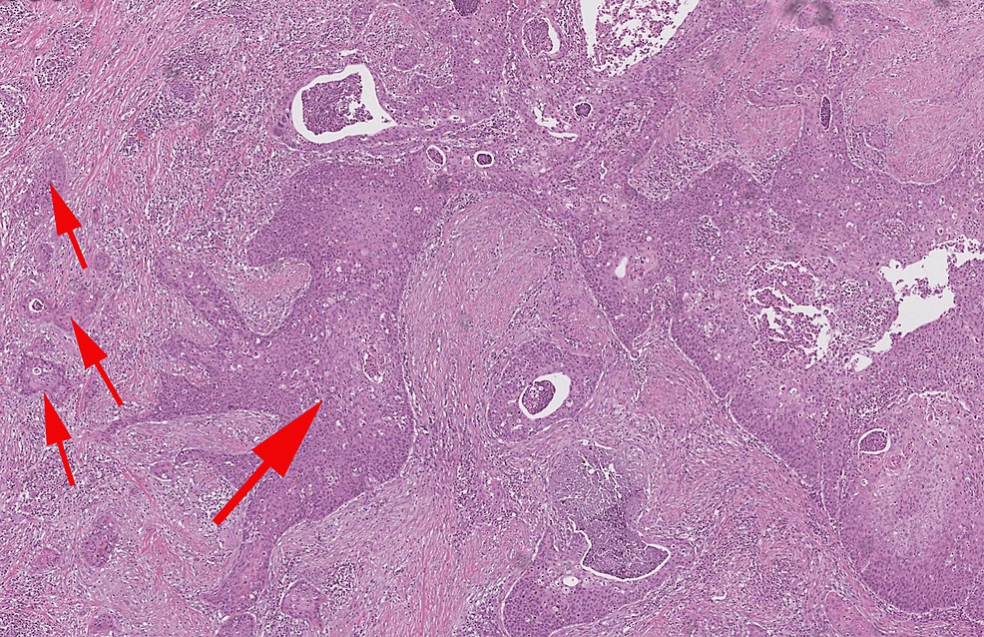
Microphotography showing an invasive carcinomatous proliferation invading the lamina propria of the glans (red arrows show invasion into the lamina propria) (HE 100X) HE: Hematoxylin & Eosin

**Figure 3:**
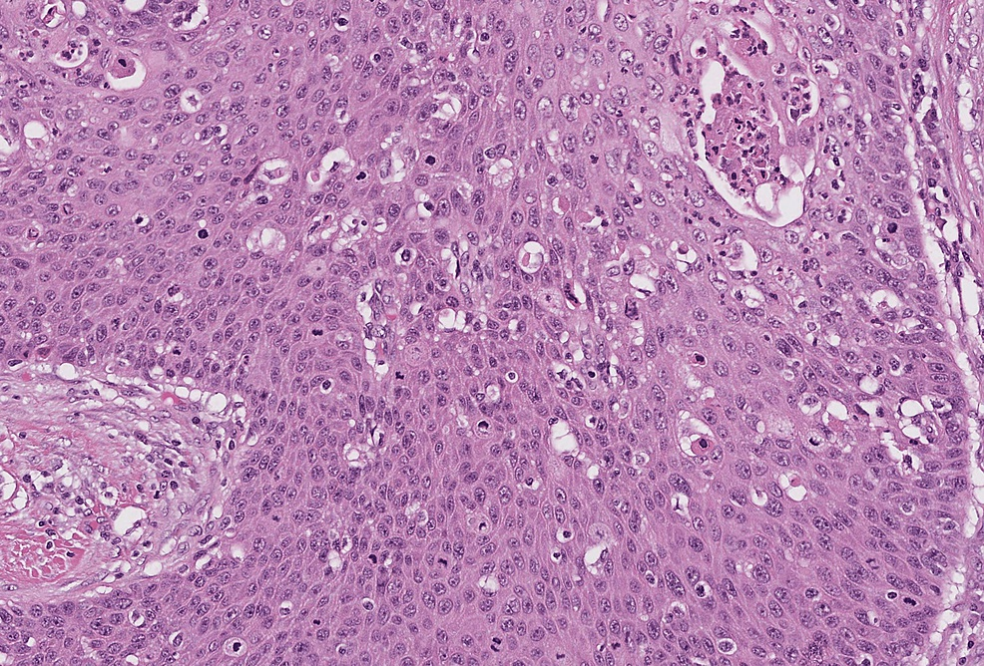
The neoplastic cells were highly atypical with evidence of keratinocyte differentiation, essentially in the form of intercellular bridges (HE 200X) HE: Hematoxylin & Eosin

Hemoglobin was normal at 12.5 g/dl, blood creatinine level was normal at 11 mg/l, and blood urea level was at 15 mg/dl. A biopsy of the lesion was performed, and a pathological examination revealed the presence of an invasive carcinomatous proliferation invading the lamina propria of the glans. The neoplastic cells were highly atypical with evidence of keratinocyte differentiation, essentially in the form of intercellular bridges. Some degree of keratinization could be appreciated, and the stroma contained many lymphocytes and plasma cells. The diagnosis of invasive keratinizing moderately squamous cell carcinoma of the glans was established (Figures 2, 3)

A thoracic, abdominal, and pelvic computed tomography scan was performed and showed bilateral inguinal lymphadenopathies with no evidence of metastatic disease. We have performed a complete penectomy with bilateral inguinal lymph node dissection. Pathological examination of the surgical specimen found the same proliferation as described in the biopsy report. The depth of invasion was measured at 9 mm, with the invasion of the corpus spongiosum. No invasion of the dartos or the corpora cavernosa was reported. Section margins were free of malignant diseases. The patient is currently receiving adjuvant chemotherapy based on cisplatin.

## Discussion

Penile cancer is a rare malignancy that classically affects elderly patients with a mean age at diagnosis of 60 years. Our reported case was 95 years old at the time of diagnosis [1]. Many risk factors have been reported, such as infection by the human papillomavirus (HPV) and the number of sexual partners, with up to 42% of cases of penile cancer being HPV associated [1]. Other risk factors include smoking, poor hygiene, and phimosis [1]. In terms of protective factors, neonatal circumcision has been reported to reduce the risk of penile cancer, in addition to the fact that this malignancy occurs almost exclusively in uncircumcised patients [1]. Circumcision prevents the occurrence of many conditions that can favor the development of penile cancer such as retention of smegma, lichen sclerosus, phimosis, and HPV infections. Our reported case was uncircumcised [1].

Diagnosis is often established after a well-documented delay. In a study conducted in Singapore, the mean delay after initial symptoms was 4 months. In our case, the delay was of 2 months [2].

The glans or the prepuce are often the initial sites for penile cancer development. Evolution is made by the local extension of the tumor to the whole gland, shaft, and corpora. The lesion can present as an ulceration, an induration, a wart-like lesion, or a painless nodule. In locally advanced cases, erosion of the prepuce with discharge occurs.

Lymphatic dissemination of penile cancer cells follows the lymphatic routes to the regional superficial and deep inguinal, femoral and iliac lymph nodes. Distant metastases can also occur during the late evolution of cases of penile cancer and are often observed in the lung, bones, and liver [3]. On the practical level, the extension of penile cancer cases can be assessed in different ways [3].

The invasion of the corpus cavernosum can be accurately determined with physical examination [4]. When an organ-sparing therapy is planned, a more precise staging using artificial erection with contrast-enhanced Magnetic Resonance Imaging (MRI) can be used [5].

Squamous cell carcinoma is the most frequently observed histological type. This histological type can be further divided into several subtypes: the usual squamous cell carcinoma (48-65%0, warty squamous cell carcinoma (7-10%), basaloid squamous cell carcinoma (4-10%), verrucous squamous cell carcinoma (3-8%), and papillary squamous cell carcinoma (5-15%) [6].

The grading plays a major role in the management of patients with penile cancer. The pathological grading for penile cancer depends on the degree of differentiation with well-differentiated cases graded as G1, moderately differentiated cases as G2, poorly differentiated cases as G3, and finally undifferentiated cases as G4 [7]. On the therapeutic level, surgical amputation remains the gold standard for the oncologic treatment of penile cancer. For Tis, Ta, and T1 stages, glans-sparing or organ-sparing techniques can be performed. A grade 1 or grade 2 histology (Good prognosis histology) is another condition for conservative therapy. In our case, deep infiltration of the penis was a clear indication of radical oncological resection. Inguinal lymph nodes’ early dissection confers a better prognosis when compared to results obtained only by surveillance or by delayed inguinal dissection [8]. Many complications are reported following inguinal lymph node dissection, and these include phlebitis and secondary pulmonary embolism, flap necrosis, wound infection, and lymphoedema of the scrotum and lower limbs [8]. Adjuvant chemotherapy can also be used for N2 disease after surgery and inguinal lymph node dissection. Chemotherapy based on cisplatin could also be suggested as neoadjuvant therapy in cases of the presence of palpable, large immobile inguinal lymph nodes.

In terms of prognostic factors, the most important one remains the presence of inguinal lymph node metastases, especially in cases of penile squamous cell carcinoma [9].

It is however important to mention that not all lymphadenopathies are of metastatic nature since 47%-85% of reported clinically detected lymphadenopathies are metastatic whereas the remaining cases are inflammatory reactions [9].

## Conclusions

Penile cancer is rare; however, it often presents with a bad prognosis since a delay in diagnosis occurs and a presentation in the late stages of the disease is encountered. The treatment is essentially based on surgical resection with penile amputation being the gold-standard surgical technique. Other organ-sparing techniques exist but are indicated only for early cancer stages. The management of lymph nodes in penile cancer remains to be a challenge, although many benefits have been reported in cases treated with early inguinal lymph node dissection. In terms of prognostic factors, the most significant one remains to be the presence of inguinal lymph node metastases since it is highly correlated with the presence of distant metastases.
